# Crystal structure of 3-meth­oxy-2-[5-(naphthalen-1-yl)-4,5-di­hydro-1*H*-pyrazol-3-yl]phenol

**DOI:** 10.1107/S2056989015018472

**Published:** 2015-10-07

**Authors:** Dongsoo Koh

**Affiliations:** a Department of Applied Chemistry, Dongduk Women’s University, Seoul 136-714, Republic of Korea

**Keywords:** crystal structure, pyrazoline, hydrogen bonds, N—H⋯π inter­action, C—H⋯π inter­action

## Abstract

In the title compound, C_20_H_18_N_2_O_2_, the central pyrazoline ring has an envelope conformation with the atom substituted by the naphthalene ring as the flap. It bridges a benzene ring and a naphthalene ring system which are almost normal to one another, making a dihedral angle of 82.03 (6) °. There is an intra­molecular O—H⋯N hydrogen bond forming an *S*(6) ring motif. In the crystal, mol­ecules are linked by pairs of N—H⋯π inter­actions, forming inversion dimers. There are also C—H⋯π inter­actions present and the dimers are linked *via* C—H⋯O hydrogen bonds, forming ribbons propagating along the *a*-axis direction.

## Related literature   

For the biological properties and synthesis of pyrazoline derivatives, see: Viveka *et al.* (2015 ▸); Neudorfer *et al.* (2014 ▸); Hwang *et al.* (2013 ▸); Congiu *et al.* (2010 ▸). For the N—H⋯π inter­action, see: Naveen *et al.* (2015 ▸). For related structures, see: Zhu *et al.* (2013 ▸); Patel *et al.* (2013 ▸).
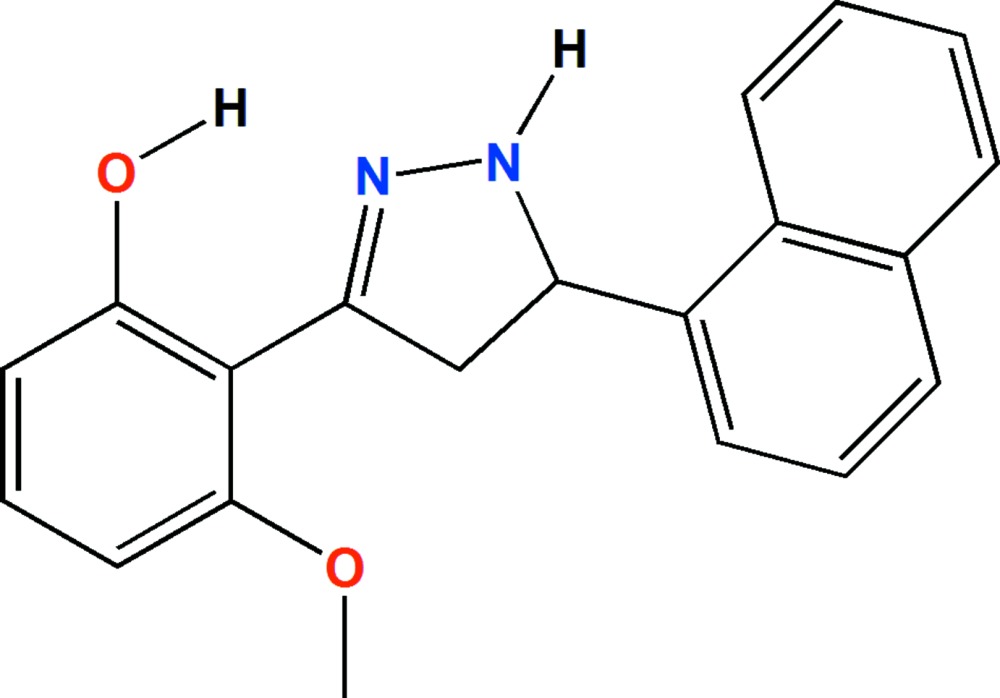


## Experimental   

### Crystal data  

C_20_H_18_N_2_O_2_
*M* *_r_* = 318.36
Triclinic, 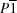

*a* = 7.7280 (12) Å
*b* = 8.6933 (14) Å
*c* = 12.721 (2) Å
α = 78.507 (4)°
β = 73.781 (4)°
γ = 76.148 (4)°
*V* = 788.7 (2) Å^3^
*Z* = 2
Mo *K*α radiation
μ = 0.09 mm^−1^
*T* = 147 K
0.23 × 0.14 × 0.10 mm

### Data collection  

Bruker Kappa APEX-DUO CCD diffractometer
Absorption correction: multi-scan (*SADABS*; Bruker, 2012 ▸) *T*_min_ = 0.980, *T*_max_ = 0.991
6731 measured reflections
3605 independent reflections
2963 reflections with *I* > 2σ(*I*)
*R*_int_ = 0.027

### Refinement  

*R*[*F* ^2^ > 2σ(*F* ^2^)] = 0.042
*wR*(*F* ^2^) = 0.119
*S* = 1.06
3605 reflections
226 parameters
H atoms treated by a mixture of independent and constrained refinement
Δρ_max_ = 0.28 e Å^−3^
Δρ_min_ = −0.26 e Å^−3^

### 

Data collection: *APEX2* (Bruker, 2012 ▸); cell refinement: *SAINT* (Bruker, 2012 ▸); data reduction: *SAINT*; program(s) used to solve structure: *SHELXS97* (Sheldrick, 2008 ▸); program(s) used to refine structure: *SHELXL97* (Sheldrick, 2008 ▸); molecular graphics: *PLATON* (Spek, 2009 ▸); software used to prepare material for publication: *SHELXTL* (Sheldrick, 2008 ▸) and *PLATON*.

## Supplementary Material

Crystal structure: contains datablock(s) I, New_Global_Publ_Block. DOI: 10.1107/S2056989015018472/su5207sup1.cif

Structure factors: contains datablock(s) I. DOI: 10.1107/S2056989015018472/su5207Isup2.hkl

Supporting information file. DOI: 10.1107/S2056989015018472/su5207Isup3.cml

**Fig. 1.**
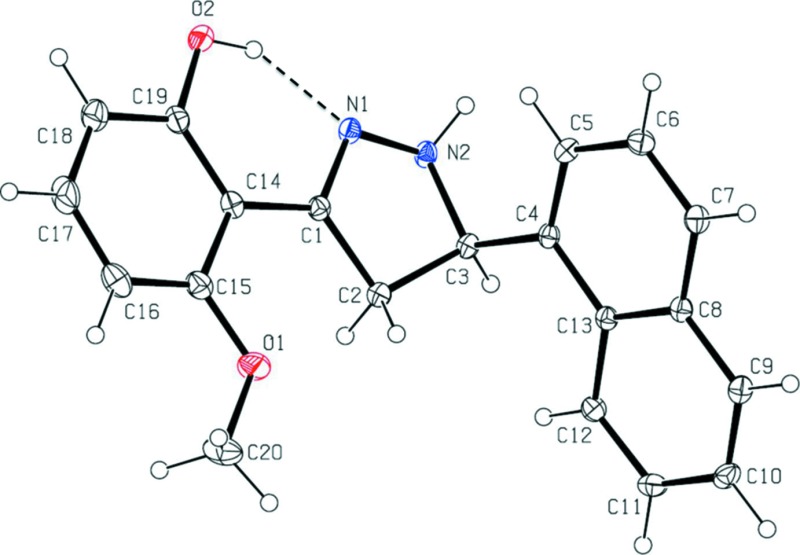
The molecular structure of the title compound, with atom labelling. Displacement ellipsoids are drawn at the 30% probability level. The intramolecular O-H···N hydrogen bond is shown as a dashed line (see Table 1).

. DOI: 10.1107/S2056989015018472/su5207fig1.tif
The mol­ecular structure of the title compound, with atom labelling. Displacement ellipsoids are drawn at the 30% probability level. The intra­molecular O-H⋯N hydrogen bond is shown as a dashed line (see Table 1).

**Fig. 2.**
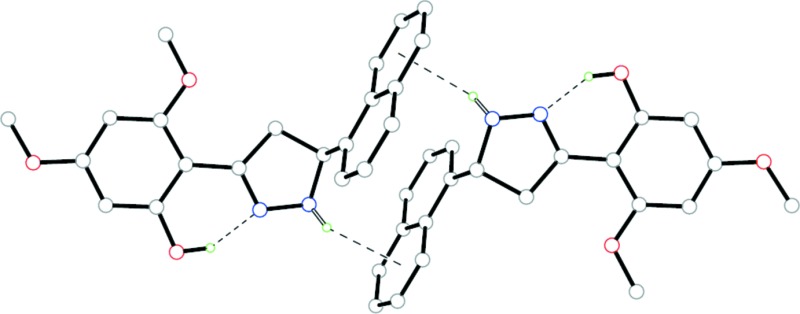
Part of the crystal structure of the title compound, showing the intramolecular O—H···N hydrogen bond and the intermolecular N—H···π interactions, as dashed lines (see Table 1). H atoms not involved in these interactions have been omitted for clarity.

. DOI: 10.1107/S2056989015018472/su5207fig2.tif
Part of the crystal structure of the title compound, showing the intra­molecular O—H⋯N hydrogen bond and the inter­molecular N—H⋯π inter­actions, as dashed lines (see Table 1). H atoms not involved in these inter­actions have been omitted for clarity.

CCDC reference: 1429221

Additional supporting information:  crystallographic information; 3D view; checkCIF report

## Figures and Tables

**Table 1:** Hydrogen-bond geometry (, ) *Cg*2 and *Cg*3 are the centroids of rings C4C8/C13 and C8C13, respectively.

*D*H*A*	*D*H	H*A*	*D* *A*	*D*H*A*
O2H2*O*N1	0.87(2)	1.79(2)	2.5681(14)	148.0(19)
N2H2*N* *Cg*3^i^	0.88(2)	2.56(2)	3.1811(13)	128.1(14)
C3H3*A* *Cg*2^i^	1.00	2.80	3.5306(15)	130
C12H12*A*O2^ii^	0.95	2.54	3.4488(17)	161

Symmetry codes: (i) 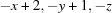; (ii) 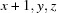.

## supplementary crystallographic information

### S1. Introduction

Pyrazolines have been reported to show a wide range of biological activities: They have been reported to be effective as Alzheimer drugs (Neudorfer *et al.*, 2014), and as having anti-inflammatory (Viveka *et al.*, 2015) and anti­tumor properties (Congiu *et al.*, 2010). The title pyrazoline derivative was synthesized in continuation of our research program (Hwang *et al.* 2013), and we report herein on its crystal structure.

The molecular structure of the title compound is shown in Fig. 1. The central pyrazoline ring (N1/N2/C1—C3) has an envelope conformation with the atom C3 as the flap. The benzene ring (C14—C19) and the naphthalene ring system (C4—C13) are attached to the central pyrazoline ring (N1/N2/C1—C3) at positions C1 and C3, respectively. The benzene and naphthalene ring are almost normal to one another with a dihedral angle of is 82.03 (6) °. The meth­oxy group at the ortho position of the benzene ring is almost coplanar with the ring [C16—C15—O1—C20 = 2.2 (2) °]. The hydroxyl group at the ortho position of the benzene ring makes an intra­molecular O—H···N hydrogen bond to form an S(6) ring motif.

In the crystal, molecules are linked by pairs of N—H···π inter­actions forming inversion dimers (Fig. 2 and Table 1). There are also C—H···π inter­actions present and the dimers are linked *via* C—H···O hydrogen bonds forming ribbons propagating along the *a* axis direction. (Table 1).

An example of inter­molecular N—H···π inter­action in pyrazoline system was reported in a recent publication (Naveen *et al.*, 2015). Examples of pyrazoline structures have been also published (Zhu *et al.*, 2013; Patel *et al.*, 2013).

### S2. Experimental

To a solution of 6-meth­oxy-2-hy­droxy­aceto­phenone (10 mmol, 1.66 g) in 40 ml of ethanol was added 1-naphthaldehyde (10 mmol, 1.56 g) and the temperature was adjusted to around 276-277 K in an ice-bath. To the reaction mixture was added 10 ml of 50% (w/v) aqueous KOH solution and the reaction mixture was stirred at room temperature for 24 h. At the end of the reaction, ice water was added to the mixture and it was acidified with 6N HCl (pH = 3-4). The resulting precipitate was filtered and washed with water and ethanol. The crude solid was purified by recrystallization from ethanol to give pure chalcone. Excess hydrazine monohydrate (1 ml of 64-65% solution, 13 mmol) was added to a solution of the chalcone compound (5 mmol, 1.52 g) in 30 ml anhydrous ethanol, and the solution was refluxed at 360 K for 5 h. The reaction mixture was cooled to room temperature to yield a solid that was then filtered. The crude solids were purified by recrystallization from ethanol to afford the title compound as yellow needles (m.p.: 429-430 K; yield: 56%).

### S2.1. Refinement

Crystal data, data collection and structure refinement details are summarized in Table 2. The NH and OH H atoms were located in a difference Fourier map and freely refined. The C-bound H atoms were fixed geometrically and allowed to ride on their parent atoms: C—H = 0.95 - 1.00 Å with *U*_iso_(H) = 1.5*U*_eq_(*C*) for methyl H atoms and 1.2*U*_eq_(C) for other H atoms.

### Crystal data

**Table:**

C_20_H_18_N_2_O_2_	*Z* = 2
*M**_r_* = 318.36	*F*(000) = 336
Triclinic, *P*1	*D*_x_ = 1.341 Mg m^−^^3^
Hall symbol: -P 1	Mo *K*α radiation, λ = 0.71073 Å
*a* = 7.7280 (12) Å	Cell parameters from 2949 reflections
*b* = 8.6933 (14) Å	θ = 2.4–27.5°
*c* = 12.721 (2) Å	µ = 0.09 mm^−^^1^
α = 78.507 (4)°	*T* = 147 K
β = 73.781 (4)°	Needle, yellow
γ = 76.148 (4)°	0.23 × 0.14 × 0.10 mm
*V* = 788.7 (2) Å^3^	

### Data collection

**Table:**

Bruker Kappa APEX-DUO CCD diffractometer	3605 independent reflections
Radiation source: fine-focus sealed tube	2963 reflections with *I* > 2σ(*I*)
Bruker Triumph monochromator	*R*_int_ = 0.027
φ and ω scans	θ_max_ = 27.5°, θ_min_ = 1.7°
Absorption correction: multi-scan (*SADABS*; Bruker, 2012)	*h* = −10→9
*T*_min_ = 0.980, *T*_max_ = 0.991	*k* = −11→10
6731 measured reflections	*l* = −16→16

### Refinement

**Table:**

Refinement on *F*^2^	Primary atom site location: structure-invariant direct methods
Least-squares matrix: full	Secondary atom site location: difference Fourier map
*R*[*F*^2^ > 2σ(*F*^2^)] = 0.042	Hydrogen site location: inferred from neighbouring sites
*wR*(*F*^2^) = 0.119	H atoms treated by a mixture of independent and constrained refinement
*S* = 1.06	*w* = 1/[σ^2^(*F*_o_^2^) + (0.0564*P*)^2^ + 0.190*P*] where *P* = (*F*_o_^2^ + 2*F*_c_^2^)/3
3605 reflections	(Δ/σ)_max_ < 0.001
226 parameters	Δρ_max_ = 0.28 e Å^−^^3^
0 restraints	Δρ_min_ = −0.26 e Å^−^^3^

### Special details

**Table:**

Geometry. All e.s.d.'s (except the e.s.d. in the dihedral angle between two l.s. planes) are estimated using the full covariance matrix. The cell e.s.d.'s are taken into account individually in the estimation of e.s.d.'s in distances, angles and torsion angles; correlations between e.s.d.'s in cell parameters are only used when they are defined by crystal symmetry. An approximate (isotropic) treatment of cell e.s.d.'s is used for estimating e.s.d.'s involving l.s. planes.
Refinement. Refinement of *F*^2^ against ALL reflections. The weighted *R*-factor *wR* and goodness of fit *S* are based on *F*^2^, conventional *R*-factors *R* are based on *F*, with *F* set to zero for negative *F*^2^. The threshold expression of *F*^2^ > σ(*F*^2^) is used only for calculating *R*-factors(gt) *etc*. and is not relevant to the choice of reflections for refinement. *R*-factors based on *F*^2^ are statistically about twice as large as those based on *F*, and *R*- factors based on ALL data will be even larger.

### Fractional atomic coordinates and isotropic or equivalent isotropic displacement parameters (Å^2^)

**Table:**

	*x*	*y*	*z*	*U*_iso_*/*U*_eq_	
O1	0.59916 (15)	0.28958 (14)	0.48006 (8)	0.0359 (3)	
O2	0.28750 (13)	0.15225 (12)	0.24649 (8)	0.0271 (2)	
N1	0.58945 (14)	0.25517 (12)	0.16583 (8)	0.0194 (2)	
N2	0.75708 (14)	0.27317 (13)	0.09019 (9)	0.0201 (2)	
C1	0.60118 (16)	0.26040 (14)	0.26482 (10)	0.0185 (3)	
C2	0.78599 (16)	0.29279 (15)	0.26322 (10)	0.0206 (3)	
H2A	0.8680	0.1942	0.2881	0.025*	
H2B	0.7727	0.3756	0.3098	0.025*	
C3	0.85658 (16)	0.35262 (14)	0.13973 (10)	0.0187 (3)	
H3A	0.9918	0.3116	0.1156	0.022*	
C4	0.81502 (15)	0.53454 (14)	0.11606 (10)	0.0173 (2)	
C5	0.67014 (16)	0.61697 (15)	0.07244 (11)	0.0207 (3)	
H5A	0.5946	0.5589	0.0550	0.025*	
C6	0.63041 (17)	0.78560 (15)	0.05276 (11)	0.0236 (3)	
H6A	0.5291	0.8395	0.0224	0.028*	
C7	0.73662 (17)	0.87181 (15)	0.07702 (11)	0.0223 (3)	
H7A	0.7090	0.9854	0.0635	0.027*	
C8	0.88810 (16)	0.79261 (14)	0.12231 (10)	0.0182 (3)	
C9	1.00239 (17)	0.88065 (15)	0.14546 (10)	0.0219 (3)	
H9A	0.9754	0.9943	0.1319	0.026*	
C10	1.15088 (17)	0.80452 (16)	0.18700 (11)	0.0246 (3)	
H10A	1.2267	0.8649	0.2018	0.029*	
C11	1.19088 (17)	0.63583 (16)	0.20777 (11)	0.0239 (3)	
H11A	1.2940	0.5830	0.2367	0.029*	
C12	1.08277 (16)	0.54708 (15)	0.18673 (10)	0.0207 (3)	
H12A	1.1113	0.4336	0.2020	0.025*	
C13	0.92878 (15)	0.62248 (14)	0.14245 (9)	0.0171 (2)	
C14	0.45128 (16)	0.22550 (15)	0.36055 (10)	0.0206 (3)	
C15	0.45112 (18)	0.23952 (16)	0.46981 (11)	0.0260 (3)	
C16	0.3089 (2)	0.20441 (19)	0.55885 (12)	0.0347 (3)	
H16A	0.3110	0.2143	0.6315	0.042*	
C17	0.1636 (2)	0.1547 (2)	0.54096 (13)	0.0389 (4)	
H17A	0.0656	0.1311	0.6021	0.047*	
C18	0.1581 (2)	0.13886 (19)	0.43663 (12)	0.0340 (3)	
H18A	0.0571	0.1046	0.4260	0.041*	
C19	0.30079 (17)	0.17304 (16)	0.34678 (11)	0.0234 (3)	
C20	0.6108 (2)	0.3009 (2)	0.58802 (13)	0.0419 (4)	
H20A	0.7229	0.3389	0.5827	0.063*	
H20B	0.6140	0.1953	0.6332	0.063*	
H20C	0.5034	0.3764	0.6224	0.063*	
H2O	0.383 (3)	0.178 (2)	0.1972 (18)	0.050 (5)*	
H2N	0.739 (2)	0.318 (2)	0.0247 (15)	0.032 (4)*	

### Atomic displacement parameters (Å^2^)

**Table:**

	*U*^11^	*U*^22^	*U*^33^	*U*^12^	*U*^13^	*U*^23^
O1	0.0432 (6)	0.0524 (7)	0.0186 (5)	−0.0193 (5)	−0.0083 (4)	−0.0069 (5)
O2	0.0268 (5)	0.0338 (6)	0.0235 (5)	−0.0144 (4)	−0.0055 (4)	−0.0010 (4)
N1	0.0224 (5)	0.0184 (5)	0.0177 (5)	−0.0075 (4)	−0.0019 (4)	−0.0034 (4)
N2	0.0237 (5)	0.0202 (5)	0.0167 (5)	−0.0086 (4)	−0.0009 (4)	−0.0042 (4)
C1	0.0216 (6)	0.0148 (6)	0.0192 (6)	−0.0041 (4)	−0.0047 (5)	−0.0027 (5)
C2	0.0218 (6)	0.0197 (6)	0.0210 (6)	−0.0053 (4)	−0.0059 (5)	−0.0022 (5)
C3	0.0186 (5)	0.0167 (6)	0.0209 (6)	−0.0045 (4)	−0.0033 (4)	−0.0036 (5)
C4	0.0175 (5)	0.0171 (6)	0.0160 (6)	−0.0046 (4)	−0.0003 (4)	−0.0033 (4)
C5	0.0190 (6)	0.0210 (6)	0.0236 (6)	−0.0063 (5)	−0.0049 (5)	−0.0045 (5)
C6	0.0209 (6)	0.0222 (6)	0.0265 (7)	−0.0004 (5)	−0.0079 (5)	−0.0021 (5)
C7	0.0235 (6)	0.0165 (6)	0.0250 (7)	−0.0019 (5)	−0.0044 (5)	−0.0033 (5)
C8	0.0196 (6)	0.0183 (6)	0.0155 (6)	−0.0042 (4)	−0.0003 (4)	−0.0046 (5)
C9	0.0260 (6)	0.0206 (6)	0.0194 (6)	−0.0074 (5)	−0.0010 (5)	−0.0066 (5)
C10	0.0267 (6)	0.0306 (7)	0.0213 (6)	−0.0126 (5)	−0.0041 (5)	−0.0089 (5)
C11	0.0209 (6)	0.0321 (7)	0.0202 (6)	−0.0041 (5)	−0.0073 (5)	−0.0055 (5)
C12	0.0213 (6)	0.0208 (6)	0.0194 (6)	−0.0035 (5)	−0.0046 (5)	−0.0029 (5)
C13	0.0177 (5)	0.0185 (6)	0.0142 (6)	−0.0039 (4)	−0.0013 (4)	−0.0032 (4)
C14	0.0242 (6)	0.0177 (6)	0.0181 (6)	−0.0033 (5)	−0.0034 (5)	−0.0013 (5)
C15	0.0306 (7)	0.0260 (7)	0.0207 (7)	−0.0052 (5)	−0.0061 (5)	−0.0024 (5)
C16	0.0419 (8)	0.0403 (9)	0.0171 (7)	−0.0072 (6)	−0.0017 (6)	−0.0017 (6)
C17	0.0331 (8)	0.0492 (10)	0.0256 (8)	−0.0127 (7)	0.0054 (6)	0.0028 (7)
C18	0.0271 (7)	0.0419 (9)	0.0301 (8)	−0.0130 (6)	−0.0030 (6)	0.0036 (6)
C19	0.0243 (6)	0.0220 (6)	0.0217 (7)	−0.0047 (5)	−0.0049 (5)	0.0013 (5)
C20	0.0533 (10)	0.0545 (11)	0.0247 (8)	−0.0106 (8)	−0.0159 (7)	−0.0118 (7)

### Geometric parameters (Å, º)

**Table:**

O1—C15	1.3637 (16)	C8—C9	1.4183 (16)
O1—C20	1.4253 (17)	C8—C13	1.4223 (17)
O2—C19	1.3582 (16)	C9—C10	1.3675 (18)
O2—H2O	0.87 (2)	C9—H9A	0.9500
N1—C1	1.2980 (16)	C10—C11	1.4108 (19)
N1—N2	1.4032 (14)	C10—H10A	0.9500
N2—C3	1.4710 (15)	C11—C12	1.3714 (17)
N2—H2N	0.880 (18)	C11—H11A	0.9500
C1—C14	1.4662 (17)	C12—C13	1.4201 (16)
C1—C2	1.5145 (16)	C12—H12A	0.9500
C2—C3	1.5387 (17)	C14—C19	1.4109 (18)
C2—H2A	0.9900	C14—C15	1.4189 (18)
C2—H2B	0.9900	C15—C16	1.383 (2)
C3—C4	1.5218 (16)	C16—C17	1.383 (2)
C3—H3A	1.0000	C16—H16A	0.9500
C4—C5	1.3709 (17)	C17—C18	1.375 (2)
C4—C13	1.4349 (16)	C17—H17A	0.9500
C5—C6	1.4097 (18)	C18—C19	1.3885 (19)
C5—H5A	0.9500	C18—H18A	0.9500
C6—C7	1.3634 (18)	C20—H20A	0.9800
C6—H6A	0.9500	C20—H20B	0.9800
C7—C8	1.4174 (17)	C20—H20C	0.9800
C7—H7A	0.9500		
			
C15—O1—C20	118.42 (12)	C8—C9—H9A	119.5
C19—O2—H2O	108.3 (13)	C9—C10—C11	119.64 (11)
C1—N1—N2	109.49 (10)	C9—C10—H10A	120.2
N1—N2—C3	108.34 (9)	C11—C10—H10A	120.2
N1—N2—H2N	110.6 (11)	C12—C11—C10	120.83 (11)
C3—N2—H2N	116.6 (11)	C12—C11—H11A	119.6
N1—C1—C14	120.08 (11)	C10—C11—H11A	119.6
N1—C1—C2	111.19 (10)	C11—C12—C13	120.89 (12)
C14—C1—C2	128.58 (11)	C11—C12—H12A	119.6
C1—C2—C3	101.19 (9)	C13—C12—H12A	119.6
C1—C2—H2A	111.5	C12—C13—C8	118.13 (11)
C3—C2—H2A	111.5	C12—C13—C4	122.86 (11)
C1—C2—H2B	111.5	C8—C13—C4	119.00 (10)
C3—C2—H2B	111.5	C19—C14—C15	117.14 (11)
H2A—C2—H2B	109.3	C19—C14—C1	120.32 (11)
N2—C3—C4	114.54 (10)	C15—C14—C1	122.53 (11)
N2—C3—C2	100.63 (9)	O1—C15—C16	123.05 (12)
C4—C3—C2	111.44 (10)	O1—C15—C14	115.53 (11)
N2—C3—H3A	110.0	C16—C15—C14	121.42 (13)
C4—C3—H3A	110.0	C15—C16—C17	119.26 (13)
C2—C3—H3A	110.0	C15—C16—H16A	120.4
C5—C4—C13	119.03 (11)	C17—C16—H16A	120.4
C5—C4—C3	122.04 (10)	C18—C17—C16	121.39 (13)
C13—C4—C3	118.91 (10)	C18—C17—H17A	119.3
C4—C5—C6	121.70 (11)	C16—C17—H17A	119.3
C4—C5—H5A	119.1	C17—C18—C19	119.76 (14)
C6—C5—H5A	119.1	C17—C18—H18A	120.1
C7—C6—C5	120.35 (11)	C19—C18—H18A	120.1
C7—C6—H6A	119.8	O2—C19—C18	116.51 (12)
C5—C6—H6A	119.8	O2—C19—C14	122.46 (11)
C6—C7—C8	120.27 (12)	C18—C19—C14	121.03 (12)
C6—C7—H7A	119.9	O1—C20—H20A	109.5
C8—C7—H7A	119.9	O1—C20—H20B	109.5
C7—C8—C9	120.87 (11)	H20A—C20—H20B	109.5
C7—C8—C13	119.64 (11)	O1—C20—H20C	109.5
C9—C8—C13	119.47 (11)	H20A—C20—H20C	109.5
C10—C9—C8	121.02 (12)	H20B—C20—H20C	109.5
C10—C9—H9A	119.5		
			
C1—N1—N2—C3	−22.01 (13)	C9—C8—C13—C12	0.77 (17)
N2—N1—C1—C14	−172.78 (10)	C7—C8—C13—C4	0.13 (17)
N2—N1—C1—C2	3.08 (13)	C9—C8—C13—C4	−178.41 (10)
N1—C1—C2—C3	15.56 (13)	C5—C4—C13—C12	−179.29 (11)
C14—C1—C2—C3	−169.03 (11)	C3—C4—C13—C12	2.02 (17)
N1—N2—C3—C4	−89.55 (12)	C5—C4—C13—C8	−0.16 (17)
N1—N2—C3—C2	30.09 (12)	C3—C4—C13—C8	−178.84 (10)
C1—C2—C3—N2	−26.20 (11)	N1—C1—C14—C19	5.35 (18)
C1—C2—C3—C4	95.64 (10)	C2—C1—C14—C19	−169.70 (11)
N2—C3—C4—C5	12.88 (16)	N1—C1—C14—C15	−175.78 (11)
C2—C3—C4—C5	−100.52 (13)	C2—C1—C14—C15	9.2 (2)
N2—C3—C4—C13	−168.47 (10)	C20—O1—C15—C16	2.2 (2)
C2—C3—C4—C13	78.13 (13)	C20—O1—C15—C14	−177.87 (13)
C13—C4—C5—C6	0.10 (18)	C19—C14—C15—O1	179.62 (11)
C3—C4—C5—C6	178.75 (11)	C1—C14—C15—O1	0.72 (18)
C4—C5—C6—C7	0.0 (2)	C19—C14—C15—C16	−0.46 (19)
C5—C6—C7—C8	0.00 (19)	C1—C14—C15—C16	−179.36 (13)
C6—C7—C8—C9	178.47 (11)	O1—C15—C16—C17	179.83 (13)
C6—C7—C8—C13	−0.06 (18)	C14—C15—C16—C17	−0.1 (2)
C7—C8—C9—C10	−178.63 (12)	C15—C16—C17—C18	0.3 (2)
C13—C8—C9—C10	−0.10 (18)	C16—C17—C18—C19	0.0 (2)
C8—C9—C10—C11	−0.32 (19)	C17—C18—C19—O2	178.98 (13)
C9—C10—C11—C12	0.06 (19)	C17—C18—C19—C14	−0.6 (2)
C10—C11—C12—C13	0.63 (19)	C15—C14—C19—O2	−178.77 (11)
C11—C12—C13—C8	−1.03 (18)	C1—C14—C19—O2	0.16 (19)
C11—C12—C13—C4	178.11 (11)	C15—C14—C19—C18	0.80 (19)
C7—C8—C13—C12	179.31 (11)	C1—C14—C19—C18	179.73 (12)

### Hydrogen-bond geometry (Å, º)

Cg2 and Cg3 are the centroids of rings C4–C8/C13 and C8–C13, respectively.

**Table:**

*D*—H···*A*	*D*—H	H···*A*	*D*···*A*	*D*—H···*A*
O2—H2*O*···N1	0.87 (2)	1.79 (2)	2.5681 (14)	148.0 (19)
N2—H2*N*···*Cg*3^i^	0.88 (2)	2.56 (2)	3.1811 (13)	128.1 (14)
C3—H3*A*···*Cg*2^i^	1.00	2.80	3.5306 (15)	130
C12—H12*A*···O2^ii^	0.95	2.54	3.4488 (17)	161

Symmetry codes: (i) −*x*+2, −*y*+1, −*z*; (ii) *x*+1, *y*, *z*.
