# Supplementary figures and images for: Crystal structure of 3-meth­oxy-2-[5-(naphthalen-1-yl)-4,5-di­hydro-1H-pyrazol-3-yl]phenol
Source: Acta Crystallogr E Crystallogr Commun. 2015 Oct 7;71(Pt 11):o828–9. doi: 10.1107/S2056989015018472 (PMC4645021; doi:10.1107/S2056989015018472)

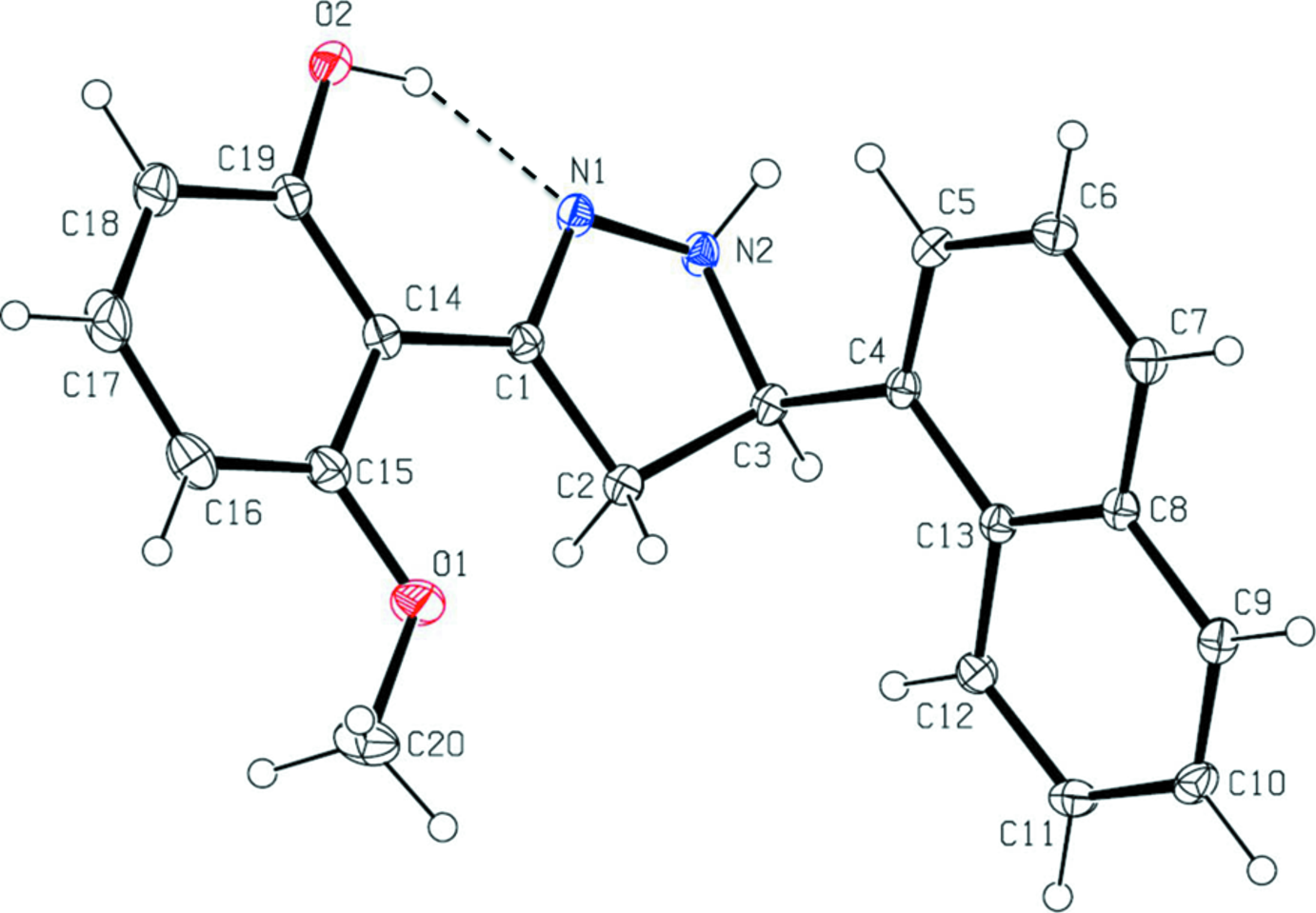

Supplement: Supplementary file 4 [file e-71-0o828-fig1.tif]

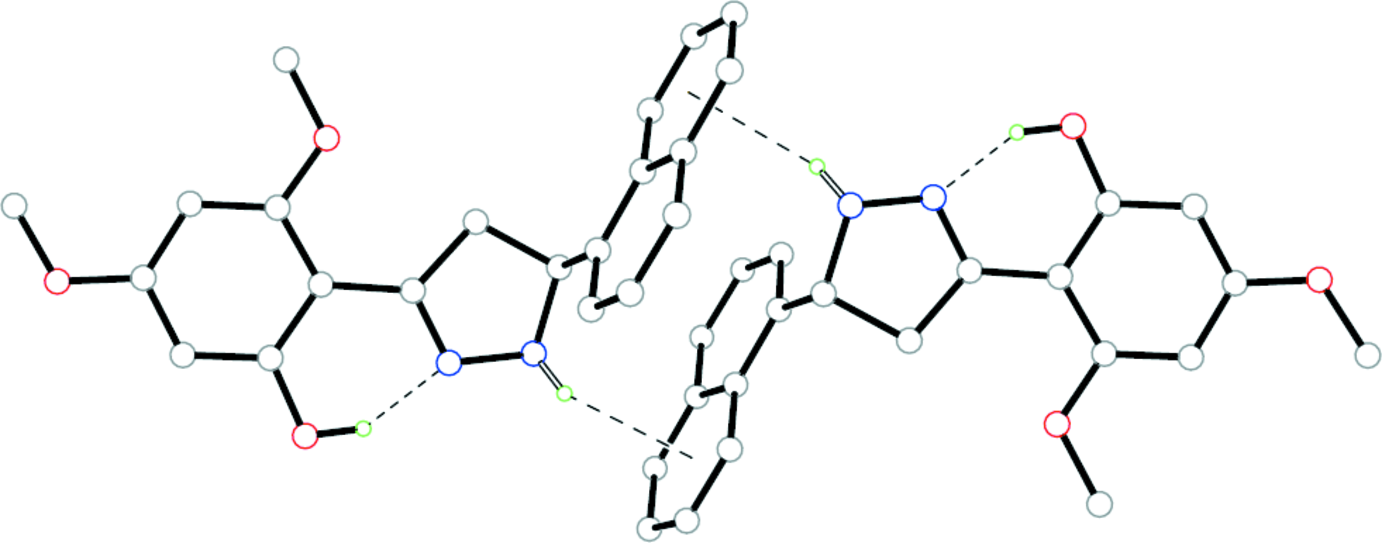

Supplement: Supplementary file 5 [file e-71-0o828-fig2.tif]
